# En-bloc Auriculectomy for Removal of a Large Pinna-Based Ear Mass in a Horse

**DOI:** 10.3389/fvets.2020.560379

**Published:** 2020-10-30

**Authors:** Auri M. Silverstone, Dane M. Tatarniuk, Elyse Durket, Alex M. Gillen

**Affiliations:** Department of Veterinary Clinical Sciences, College of Veterinary Medicine, Ames, IA, United States

**Keywords:** horse, equine, ear, aural neoplasia, auriculectomy, ear amputation, pinna

## Abstract

A 15-year-old Pony of America (POA) gelding presented for evaluation of a large mass present on the right external pinna. Based on gross appearance, the right ear mass was suspected to be neoplastic. The most likely differential diagnosis was that of a fibroblastic sarcoid. Complete auriculectomy via use of a constricting latex-tourniquet performed under multimodal analgesia was proposed as an option to achieve complete resolution of mass growth and improve patient comfort. Benefits of latex tourniquet constriction included immediate lack of bleeding associated with amputation, gradual ischemic necrosis and sloughing of tissue distant to the site of constriction, and cost-effective application. The external pinna sloughed 3 weeks following application of the constricting latex tourniquet. Complete healing was achieved within 3 months from the time of tourniquet application. The middle ear canal sealed closed as a result of auriculectomy, with no observed long-term discomfort or morbidity aside from reduction in hearing. This is the first report of total external ear amputation in the horse. Complete auriculectomy via use of a constricting latex tourniquet is a feasible method for en-bloc removal of large, complicated ear masses.

## Introduction

The ear (*Organon auditus*) of the horse is an uncommon location for development of pathology or disease, and even less commonly requires surgical intervention. Anatomically, the ear is subdivided into three divisions: external, middle, and internal regions. The external ear (*Auris externa*) consists of the funnel shaped pinna/auricula that facilitates sound wave propagation. The external ear canal is separated from the middle and inner ear regions by the tympanic membrane. The middle and inner ear canals are in close relation to the temporohyoid articulation, and contain a large diverticulum known as the guttural pouch. The external pinna is predominately composed of the auricular and annular cartilage. The pinna is sparsely covered with soft tissue consisting of a minimal amount of subcutaneous fat and the overlying skin. The external pinna connects directly to the temporal bone of the skull and can move in multiple directions in response to auditory noise or touch via the use of several extrinsic and intrinsic muscles (1).

Development of pathologic masses on the outer or inner surface of the external pinnae is uncommon. Previously reported pathologic masses include papilloma, sarcoid, melanoma, schwannoma peripheral nerve sheath tumors, adenocarcinoma, squamous cell carcinoma, inflammatory polyps, and granuloma masses secondary to auricular chondritis (2–9).

Various surgical techniques to removal pinnae-based pathological masses are available, and include direct sharp excision, sharp excision following lateral wall resection, modified vertical ear canal ablation, carbon dioxide laser excision, and cryotherapy (7–10). Re-occurrence following sharp debridement or carbon dioxide laser removal can occur. Re-occurrence following surgery may be influenced by poor surgical exposure allowing insufficient resection margins or surgical hesitancy with the aim of maintaining a cosmetic appearance (8). Non-surgical treatments include immunotherapy, topical immune modulation, topical chemotherapy, intra-lesional chemotherapy, and radiotherapy (11).

Surgical intervention of the pinnae requires an effective strategy for implementing local analgesia, as the pinnae region can be sensitive to touch and stimulation in many horses. Many procedures of the equine head and neck can now be performed using standing, sedated restraint. Regardless of surgical route, observation for post-operative pain in horses can be a challenge and requires astute observation of behavior and close monitoring of vital parameters.

This case report describes presentation and treatment of a large mass located on the inner surface of the external pinna of a horse via en-bloc auriculectomy using a latex tourniquet. Additionally, post-operative healing of the base of the ear following amputation is detailed.

## Case Report

### Case History

Written informed consent was obtained from the owners for the participation of their animal in this case report. A 15-year-old Pony of America (POA) gelding presented for evaluation of a large mass present on the right external pinna. The pony was recently purchased from a sales auction with no disclosed history, so the chronicity of the mass was unknown to the present owner.

### Clinical Findings

**Figure 1:**
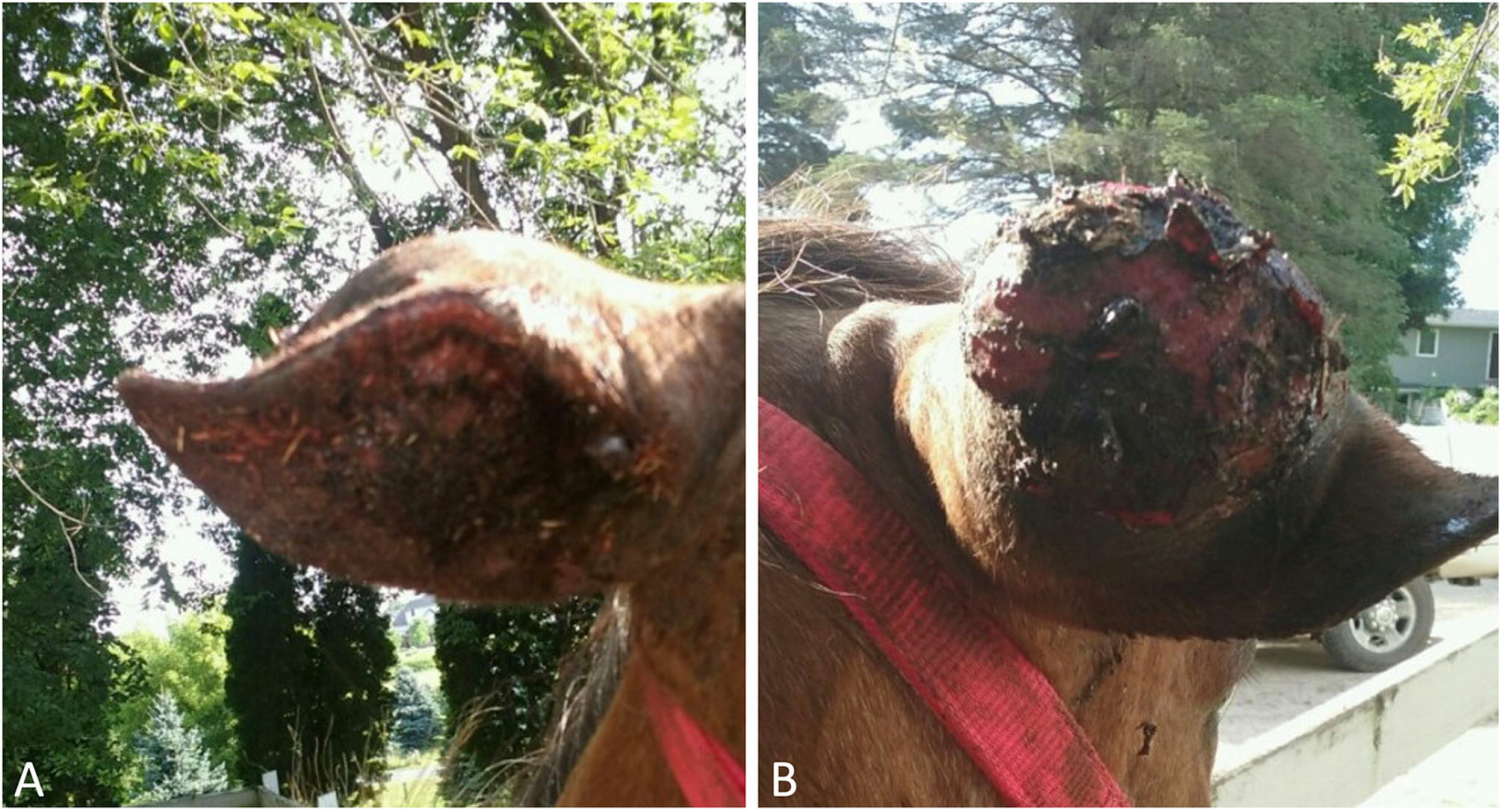
Right pinnae mass. **(A)** Rostral view; **(B)** caudal view.

The mass on the right external pinna was raised, firm, diffusely red to black, partially ulcerated with moderate crusted serous discharge (Figures 1A,B). The mass, measuring 7.6 cm in diameter, extended through the pinna and could be visualized on both the outer and inner aspect of external ear. The ear retained movement in reaction to noise and external stimulus however was ventrally deviated due to increased weight from the mass. There were no external behavioral symptoms of pain or aversion when palpating the ear or the mass. Systemically, the horse was bright, alert, responsive, in good body condition and normal weight (415 kg). It was reported by the owner that flies were excessively attracted to the location of the mass, causing irritation, and head shaking by the horse. Based on gross appearance, the right ear mass was suspected to be neoplastic. The most likely differential diagnosis was that of a fibroblastic sarcoid. Other differentials included melanoma, squamous cell carcinoma, mast cell tumor, or papillomatosis. Pre-surgical biopsy was offered but declined due to financial limitations from the owner.

### Treatment

Due to the large size of the mass, as well as abnormal pathology noted on both the external and internal aspect of the external pinna, circumferential removal at the interface of mass to normal skin was anticipated to leave a non-cosmetic, large circular fistula within the pinna. Concern was forecasted that this could also risk poor excisional margins of the mass. Complete auriculectomy was proposed as an option to achieve complete resolution of mass growth and improve patient comfort.

Use of a constricting latex-tourniquet was proposed as a method to perform total auriculectomy. The latex tourniquet is applied using a bander device^1^. Benefits of latex tourniquet constriction for excision of appendages in animals include immediate lack of bleeding associated with amputation, gradual ischemic necrosis and sloughing of tissue distant to the site of constriction, lack of discomfort when applied at time of surgery or in the post-operative phase, and cost-effective application (12, 13). Based on the authors' positive experiences using latex tourniquet constriction for partial phallectomy in horses, for providing both an effective amputation and being well-tolerated by the patient, this option was discussed in detail and consented to by the owner.

**Figure 2:**
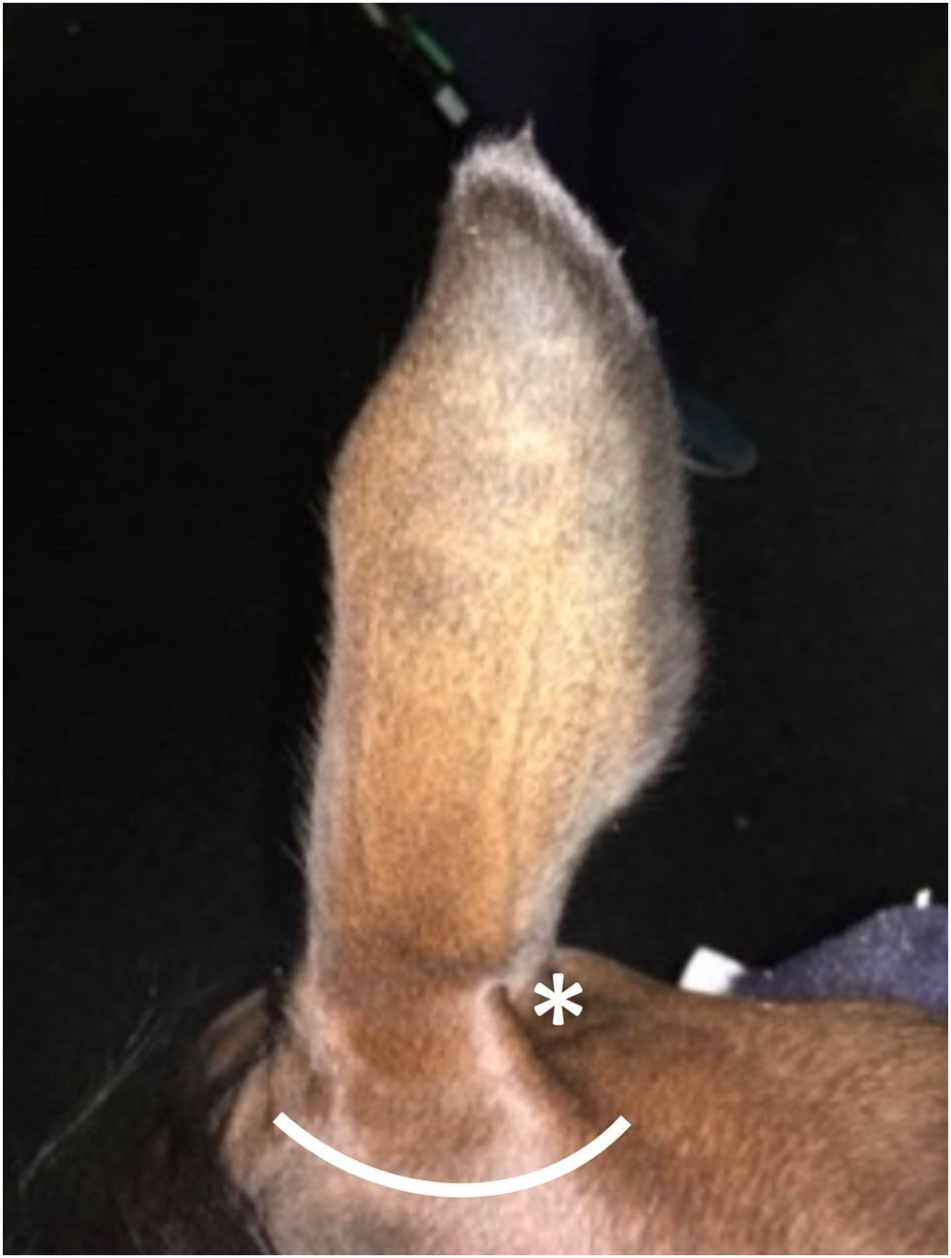
Representative picture of a normal horse demonstrating locations for injection of local anesthetics. The asterisk (*) along the caudal aspect of the pinnae denotes the location of the auriculopalpebral nerve. The curvilinear line along the lateral aspect of the pinnae denotes the approximate location of a hemi-circumferential ring-block.

The patient was sedated with detomidine^2^ (0.02 mg/kg IV; Dormosedan) and butorphanol^3^ (0.2 mg/kg IV; Torbugesic). Phenylbutazone^4^ (2 g IV; Vetribute) was administered for analgesia. Intra-muscular tetanus toxoid vaccine^5^ was administered as prophylaxis. The internal auricular nerve was individually blocked by palpating the divot within the lateral aspect of the base of the auricular cartilage, wherein 3 ml of 2% mepivacaine (Carbocaine see text footnote 5) was deposited (14). The greater auricular nerve was individually blocked by palpating the caudal aspect of the pinna to identify the nerve, and depositing 3 ml of 2% mepivacaine peri-neurally (14). An additional 10 ml of 2% mepivacaine was injected subcutaneously in a hemi-circumferential fashion along the lateral base of the ear to ensure total regional anesthesia occurred (Figure 2). Prior to placement of the latex tourniquet, skin was lightly probed and pinched with a mosquito hemostat to ensure that proper analgesia of the region had been achieved.

**Figure 3:**
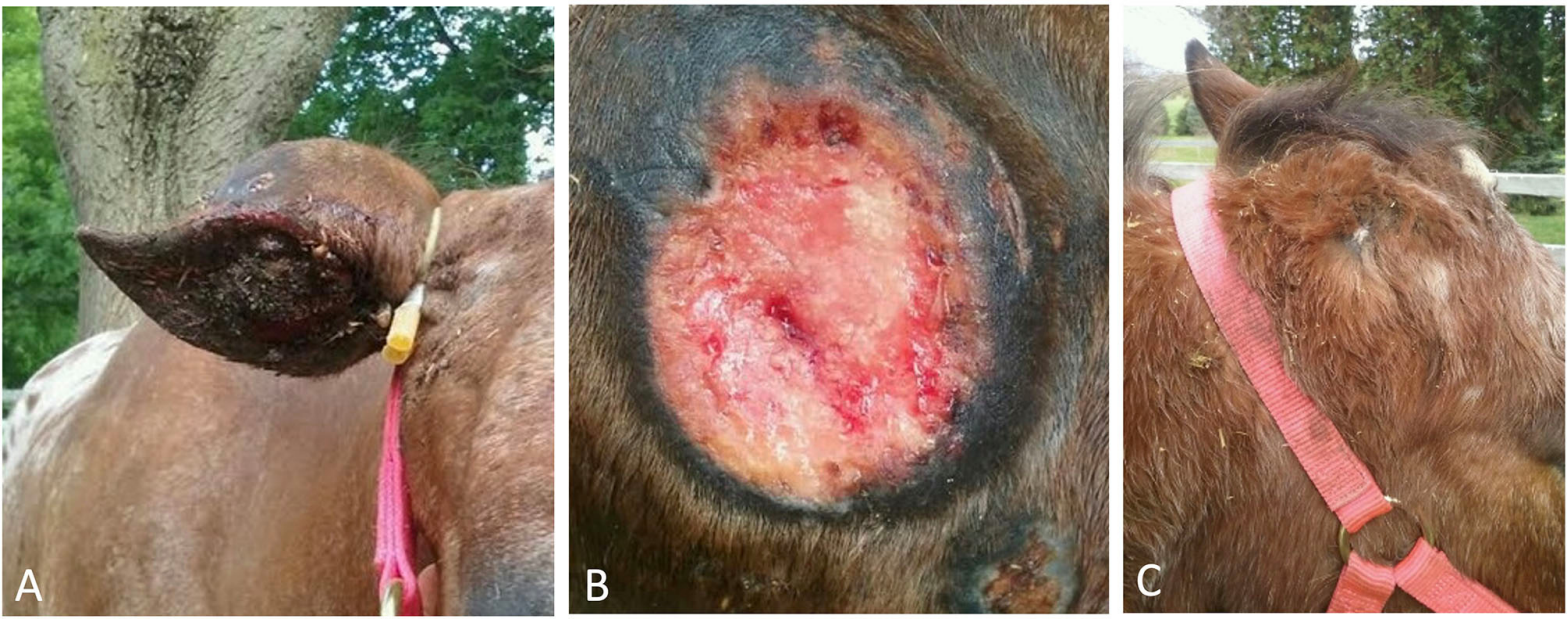
**(A)** Placement of the constricting latex tourniquet on the base of the right pinna immediately after application (rostral view); **(B)** wound at 47 days post-tourniquet application (26 days following sloughing of ear), with healing granulation bed and evidence of the middle ear canal sealing over (rostral to right of image); **(C)** healed auriculectomy with mild scar formation (rostral to right of image).

**Figure 4:**
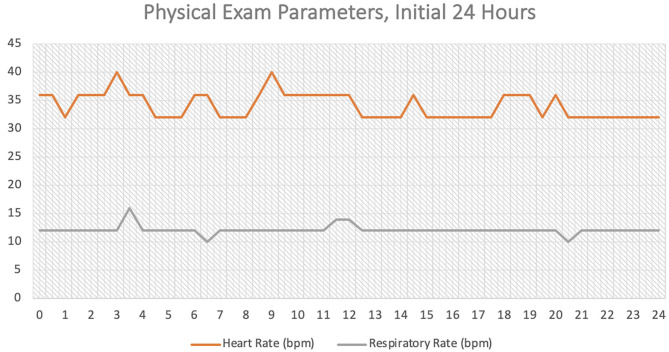
Physical exam parameters (heart rate [beats/min] and respiratory rate [breath/min]) for the initial 24 h following application of the tourniquet.

The constricting latex tourniquet was applied using the Callicrate bander device as close to the base of the ear as possible, ensuring the tourniquet was situated at least 1 cm beyond the margins of the mass (Figure 3A). The tourniquet is a latex-tubing in a dual-loop pattern with a central metal clamp. The tourniquet was maximally compressed against the ear using a ratchet mechanism, and then held in compression by crimping the central metal clamp, as performed for bovine castration or equine partial phallectomy (12, 13). The free edges of the tourniquet were then trimmed short to avoid the potential of the tourniquet irritating the face of the horse. The horse tolerated placement of the latex tourniquet under sedated restraint and local block without display of resentment or agitation. Post-operative phenylbutazone (see text footnote 4) (1 gram PO, q. 12 h, for 5 days) and long-acting ceftiofur^6^ (6.6 mg/kg IM; Excede day 0 and day 4) was administered. The horse was closely monitored for the first 24 h (regular visual and physical examinations every 30 min) by the attending surgeon or resident-in-training veterinarian to ensure no signs of marked discomfort, agitation or other behavioral symptoms of pain (pawing, head-shaking, etc.) that would have necessitated additional pain control or potential removal of the latex tourniquet. No abnormalities based on visual or physical examination were noted, which included the time period following cessation of the mepivacaine local block. For all examinations within the first 24 h, heart rate and respiratory rate were normal (ranges: 28–48 beats per minute and 12–20 breathes per minute, respectively, Figure 4). The horse maintained a normal appetite and willingness to interact with caretakers.

### Outcome

The owner was instructed on how to observe for symptoms of marked discomfort, agitation or other behavioral symptoms of pain prior to discharge. Specifically, the owner was requested to contact her first opinion veterinarian if depression, reluctance to eat, a marked reaction to noise, a head tilt, head shaking, head/face rubbing, or excessive head movements were observed. The horse was examined daily by the owner to monitor the constricted pinna as well as continued constriction of the latex tourniquet. The pinna and tourniquet were noted to have sloughed exactly 21 days following application of the latex tourniquet. The remaining base of the pinna had development of healthy granulation tissue and a narrowed orifice of the middle ear canal still evident. Gentamicin ointment, formulated for ear infections in dogs, was administered into the ear canal. The owner was instructed to clean the open wound with sterile saline and continue to apply gentamicin otic (0.03% gentamicin; Gentazol) ointment^7^ q. 12 h.

The horse was re-examined 47 days post-tourniquet application (26 days following sloughing of the external pinna) and the granulation bed was noted to have sealed over the middle ear canal (Figure 3B). Due to the unknown influence of a sealed ear canal and the potential for otitis interna or media, the granulation bed was sharply debrided under standing sedation to re-establish an orifice. Fifty-seven (57) days following tourniquet application (36 days following sloughing of ear) the base of the ear surrounding the granulation bed was moderately swollen and painful to the touch, consistent with cellulitis. The granulation bed was again noted to have sealed over the middle ear canal. The granulation bed was treated with a combined nystatin (100,000 units/ml), neomycin sulfate (2.5 mg/ml), thiostrepton (2,500 units/ml), and triamcinolone acetonide cream (1.0 mg/ml)^8^, which was repeated once daily for 5 days by the owner. Additionally, a 14-day course of trimethoprim-sulfa^9^ (25 mg/kg PO q. 24 h; Equi-sul) was prescribed. No further attempts were made to disrupt the granulation bed and re-open the middle ear canal.

The horse was re-examined 5 days later (63 days following tourniquet application, 41 days following sloughing of ear). Symptoms of swelling and pain to palpation were no longer present. The granulation bed appeared to be healing well with continued epithelization occurring circumferentially. The owner was contacted by phone at 8 and 18 months post-operatively. The owner reported that the amputation site had completely healed with minor scar formation present (Figure 3C). No further history of otitis interna, media or other abnormalities of the otorhinolaryngology system had occurred. Enough of a pronounced healed amputation stump remained to allow an equine halter to stay situated on the horse for handling. The horse could be used for trail riding, using a traditional bridle, without limitation. Follow up computed tomography (CT) was discussed with the owner as an option to evaluate the middle and inner ear, following sealing of the canal, but was declined.

## Discussion

Complete auriculectomy has been previously reported in other species, however, is infrequently performed if less invasive techniques are considered feasible (15, 16). Use of a latex-tourniquet is most commonly utilized for castration of immature and mature bulls. It has also been utilized to perform standing partial phallectomy in horses (12, 13). The technique is humane and well-tolerated when applied under local analgesia (12).

Local analgesia is imperative to patient welfare in performing this technique. Innervation of the ear derives from four nerves. The *greater auricular nerve*, a branch extending from the second cervical nerve, provides sensory information to the outer and inner surface of the pinnae. The *internal auricular nerve*, a branch of the facial nerve (cranial nerve VII) and the *auricular branch of the vagus nerve* (cranial nerve X), connect within the facial canal of the petrous temporal bone and exit through the stylomastoid foramen, providing sensory innervation to the external acoustic meatus. Lastly, the *auricular branch of the trigeminal nerve* (cranial nerve V) provides sensory innervation to the external pinnae. The internal auricular nerve and auricular branch of the trigeminal nerve run along the lateral recess of the guttural pouch, deep to the parotid salivary gland (1). In this case, local analgesia of the greater and internal auricular nerves was performed along with a hemi-circumferential ring block, under sedated restraint. This provided excellent analgesic support for application of the latex-tourniquet. For this case, mepivacaine hydrochloride (an amino amide, which inhibits influx of sodium across axonal membranes) was used. Mepivacaine hydrochloride has a reported duration ranging from 120 to 240 min (17). Potentially, the use of another local anesthetic, such as bupivacaine (duration range of 180–600 min) could have imparted a longer duration of analgesia (17). The pathophysiology of constriction ischemia and timeline for development of denervation is unknown in animals. In thermal ischemic insult (i.e., frostbite), absence of tissue sensation develops within 24–72 h of deep thermal ischemic insult (18).

The decision to pursue latex-tourniquet amputation of the external ear, as well as the uncertainty of patient comfort, was discussed in detail with the owner prior to performing this technique. Leaving the mass *in-situ* was not considered a reasonable option due to presence of significant discharge and ulceration of the skin. Additionally, removal was advocated to avoid development of other potential complications, such as neuropraxia (CN VII or CN VIII), chronic headshaking, or metastasis. Consideration of this technique as a possible option was supported by positive experiences of the co-authors utilizing latex-tourniquet amputation for partial phallectomy, as reported by Arnold et al. (13). Removal of the latex-tourniquet was planned if any post-operative discomfort were to be observed following local analgesia subsiding. Other than the brief period of discomfort 57 days post-operatively, this horse displayed no behavioral or physical examination abnormalities to suggest discomfort at any point during tourniquet application or thereafter.

Regarding analgesia, one limitation of this case is the lack of a defined pain scale to discern whether analgesic mitigation was sufficient. Inclusion of a pain scale (i.e., Horse Grimace Scale) would have more objectively and accurately determined whether adequate analgesia was provided (19). Evaluation of heart rate and respiratory rate during the first 24 h while under veterinary care did not reveal any major trend upwards in parameters. Another limitation is that the patient was discharged to the owners care after 24 h due to financial constraints limiting longer hospitalization. While many horse owners are attentive to their animals well-being, frequent physical examinations were not performed and the horse was only periodically re-evaluated by a veterinarian on-farm. The owner could have potentially missed subtle symptoms of pain or discomfort in their animal.

Following sloughing of the external ear and the latex tourniquet falling off, a healthy open granulating wound was present. However, over time, the granulation bed sealed over the ear canal. This was unexpected by the authors, however not unsurprising in hindsight given the healing pattern of other granulation wound beds associated with stomas, such as temporary tracheotomy or temporary perineal urethrostomy sites. Although the auricular cartilage within the base of the pinna is rigid, the constricting influence of the latex tourniquet for several weeks duration may have deformed the area and increased the likelihood of the canal sealing over. Signs of residual otitis externa and/or media/interna were suspicious at 36 days following sloughing of the external ear, however treatment with local and systemic antimicrobials successfully resolved external symptoms. Following this single episode, there were no further external indications of otitis media or interna. However, as the pharynx and guttural pouches were not thoroughly evaluated post-operatively, a final conclusion of absence/presence of otitis media or interna could not be definitively made. Equine are an anatomically unique species due to the presence of guttural pouches, which are extensive diverticula expanding the eustachian tube system (or, middle and inner canal). Potentially, the tympanic membrane may have become disrupted and allowed facilitated drainage into the guttural pouch system (1). Unfortunately, follow up pharyngeal and guttural pouch endoscopy to explore for evidence of internal drainage of infection was not an option.

In conclusion, en-bloc auriculectomy of the external pinna using a constricting tourniquet technique under multimodal analgesia is a possible surgical intervention for large pinna-based ear masses. This technique may be most suitable when financial limitations preclude the use of other established therapies for aural neoplasia. To the authors' knowledge, this is the first report of en-bloc (total) pinnae amputation in the horse. Following amputation, uncomplicated closure of the ear canal may develop.

## Data Availability Statement

The raw data supporting the conclusions of this article will be made available by the authors, without undue reservation.

## Ethics Statement

Written informed consent was obtained from the owners for the participation of their animal in this case report.

## Author Contributions

All authors contributed meaningfully to case management, manuscript preparation, writing, and editorial oversight of the final manuscript.

## Conflict of Interest

The authors declare that the research was conducted in the absence of any commercial or financial relationships that could be construed as a potential conflict of interest.

## Footnotes

^1^SMART Callicrate Bander®, St. Francis, KS, USA.

^2^Dormosedan, Zoetis US, Parsippany-Troy Hills, NJ, USA.

^3^Torbugesic, Zoetis US, Parsippany-Troy Hills, NJ, USA.

^4^Vetribute, VetOne, Boise, ID, USA.

^5^Carbocaine-V, Zoetis US, Parsippany-Troy Hills, NJ, USA.

^6^Excede, Zoetis US, Parsippany-Troy Hills, NJ, USA.

^7^Gentazol, VetOne, Boise, ID, USA.

^8^EnteDerm, VetOne, Boise, ID, USA.

^9^Equisul-SDT, Aurora Pharmaceutical, Northfield, MN, USA.

## References

[1] Sisson S Grossman DJ Equine head. In: Sisson S, Grossman DJ, editors. Anatomy of Domestic Animals. 4th ed London: Saunders (1953). p. 322–5.

[2] Stewart WC Baker GJ Aural squamous cell carcinoma in the horse: a case report. Equine Vet J. (1975) 7:210–11. 10.1111/j.2042-3306.1975.tb03273.x

[3] Fjordbakk CT Kennedy DG Rodriguez-Palacios A Keller S Stalker M. Inflammatory aural polyp in a horse. Can Vet J. (2006) 47:65–6. 10.4141/cjas67-009 16536231 PMC1316124

[4] Bowers JR Slocombe RF. Auricular chondrosis in a horse. Aust Vet J. (2009) 87:219–21. 10.1111/j.1751-0813.2009.00427.x 19489778

[5] Torres SM Malone ED White SD Koch SN Watson JL. The efficacy of imiquimod 5% cream (Aldara®) in the treatment of aural plaque in horses: a pilot open-label clinical trial. Vet Derm. (2010) 21:503–9. 10.1111/j.1365-3164.2009.00877.x 20500494

[6] Ahmadi N Oryan A Ghane M Geramizadeh B Schwannoma of the external auditory canal in a filly: a case report. J Eq Vet Sci. (2013) 33:1012–5. 10.1016/j.jevs.2013.02.007

[7] Skärlina EM Tvedten HW Roberts VL Gorvy DA Resection of a ceruminous adenocarcinoma in a horse by a modified vertical ear canal ablation. Eq Vet Educ. (2015) 27:462–6. 10.1111/eve.12421

[8] McCauley CT Hawkins JF Adams SB Fessler JF. Use of a carbon dioxide laser for surgical management of cutaneous masses in horses: 32 cases (1993–2000). J Am Vet Med Assoc. (2002) 220:1192–7. 10.2460/javma.2002.220.1192 11990967

[9] Lane JG. The treatment of equine sarcoids by cryosurgery. Eq Vet J. (1977) 9:127–33. 10.1111/j.2042-3306.1977.tb04003.x 891516

[10] Zwingenberger A Parks A Downs M Lateral ear canal resection and segmental pinnal excision in a horse to remove a sarcoid. Eq Vet Educ. (2002) 14:230–3. 10.1111/j.2042-3292.2002.tb00177.x

[11] Hewes CA Sullins KE Review of the treatment of equine cutaneous neoplasia. In: Proceedings of the 55th Annual Convention of the American Association of Equine Practitioners. Vol. 55 Las Vegas (2009). p. 387–93.

[12] Stafford KJ Mellor DJ. The welfare significance of the castration of cattle: a review. New Zealand Vet J. (2005) 53:271–8. 10.1080/00480169.2005.36560 16220117

[13] Arnold CE Brinsko SP Love CC Varner DD. Use of a modified Vinsot technique for partial phallectomy in 11 standing horses. J Am Vet Med Assoc. (2010) 237:82–6. 10.2460/javma.237.1.82 20590499

[14] McCoy AM Schaefer E Malone E How to perform effective blocks of the equine ear. In: Proceedings of the 55th Annual Convention of the American Association of Equine Practitioners. Vol. 53. Orlando, FL (2007). p. 397–8.

[15] Dean NR White HN Carter DS Desmond RA Carroll WR McGrew BM Outcomes following temporal bone resection. Laryngoscope. (2010) 120:1516–22. 10.1002/lary.20999 20641083 PMC3951338

[16] Schwab TM Popovitch C DeBiasio J Goldschmidt M. Clinical outcome for MCTs of canine pinnae treated with surgical excision (2004–2008). J Am Animal Hosp Assoc. (2014) 50:187–91. 10.5326/JAAHA-MS-6039 24659731

[17] Heavner JC Local anaesthetics. In: Thuron JC, Tranquiilli WJ, Genson JG, editors. Lumb and Jones' Veterinary Anaesthesia. 3rd ed Baltimore, MD: Lea and Febiger (1996). p. 30–6.

[18] Suri ML Vijayan GP Puri HC Barat AK Singh N. Neurological manifestations of frost-bite. Indian J Med Res. (1978)67:292–9. 680886

[19] Dalla Costa E Minero M Lebelt D Stucke D Canali E Leach MC. Development of the Horse Grimace Scale (HGS) as a pain assessment tool in horses undergoing routine castration. PLoS ONE. (2014) 9:e92281. 10.1371/journal.pone.0092281 24647606 PMC3960217

